# Misophonia in Tic Disorders and Their Neuropsychiatric Associations

**DOI:** 10.1002/mdc3.70017

**Published:** 2025-02-19

**Authors:** Arjun Balachandar, Talyta Cortez Grippe, Wei Kang Lim, Irene A. Malaty, Anthony E. Lang, Christos Ganos

**Affiliations:** ^1^ Division of Neurology, Department of Medicine University of Toronto Toronto Ontario Canada; ^2^ Krembil Research Institute Toronto Ontario Canada; ^3^ Edmond J. Safra Program in Parkinson's Disease and Morton and Gloria Shulman Movement Disorders Clinic, Toronto Western Hospital, UHN, Division of Neurology Department of Medicine, University of Toronto Toronto Ontario Canada; ^4^ Department of Neurology University of Florida, Fixel Institute for Neurological Diseases Gainesville Florida USA

**Keywords:** misophonia, tic disorders, neuropsychiatric disorders, functional neurological disorders

Tics are repetitive and intrusive movements, including vocalizations, typically characterized by premonitory urge with associated relief.^1^ People with tic disorders have reported heightened sensitivity to sensations, including auditory stimuli, aggravating tics.^2^ Misophonia is a form of auditory hypersensitivity characterized by severe dislike of specific sounds with intense emotional responses.^3^ Although misophonia is a well‐established phenomenon in autism spectrum disorders (ASD) and other neuropsychiatric disorders such as obsessive‐compulsive disorder (OCD),^3^, ^4^ its interaction with tic disorders remains unacknowledged. We present 6 cases illustrating the clinical spectrum of misophonia‐associated tics and their neuropsychiatric associations.

The first case was an 18‐year‐old patient with chronic tic disorder (CTD), ASD, and OCD. He had motor and vocal tics, including coprolalia (the “F‐word”) triggered by specific sounds. Misophonia to swallowing and mouth sounds, especially from his mother, triggered intense distress and tics. Avoiding proximity to his mother while eating significantly decreased misophonia and tics. Similarly, the second case was a 32‐year‐old woman with CTD, ASD, and depression, with misophonia to chewing that worsened all tics, whereas the third case was a 26‐year‐old woman with OCD, notably without ASD, and chewing misophonia that triggered violent thoughts and facial and shoulder shrugging tics.

The fourth case was a 14‐year‐old boy with CTD, ASD, and OCD. Despite tolerance to most noxious sounds, hearing the specific words “right” and “sorry” was so disturbing that rapid neck extension, aggressive head nodding, and trunk flexion tics occurred in intense bouts. Volume, tone, and word context were not modifying factors apart from greater impact with maternal origin of speech.

The fifth case was a 27‐year‐old man with CTD, OCD, ASD, and chromosome 2p16.3 deletion. Motor tics began at age 11, later developing prominent misophonia and hyperacusis with most sounds perceived as painful, including speech and environmental sounds like air‐conditioning humming (Video 1, Segment 1). He required almost‐constant noise‐canceling headphones, causing extreme social isolation and impairment. Audiology testing was unrevealing. Cognitive behavioral therapy mildly improved misophonia.

**Video 1:** Segment 1, case 4: functionally incapacitating misophonia combined with hyperacusis. Constant usage of noise‐canceling headphones over earplugs as coping method. Segment 2, case 5: functional tics triggered by misophonia to subtle noises, including coughing (the examiner), a dripping faucet, and phone notifications.

The sixth case was a 19‐year‐old woman with a functional tic disorder. She had stereotyped repetitive shouting of phrases, including “who makes the noise?” and “stop it!,” triggered by subtle background noises like breathing (including from examiners), squeaking chair noises, and door opening (Video 1, Segment 2).

These cases illustrate the range and severity of misophonia in CTDs and their neuropsychiatric associations, as well as in functional tics. Misophonia can occur with any sound but often with oral or environmental sounds^3^ and can be a debilitating symptom, commonly associated with neurodevelopmental and neuropsychiatric conditions.^3^, ^4^ Indeed, patients with primary tic disorders here also had comorbid ASD or OCD. Distinguishing their contribution from CTDs itself is challenging, but awareness that misophonia may be a tic‐modifying factor is prudent for both education and behavioral therapies. Importantly, most patients experienced misophonia that itself could trigger tics, whereas avoiding misophonia triggers resulted in the absence of or decreased tics. Abrupt emotional dysregulation may cause misophonia^5^; however, the pathophysiology remains unclear.^4^ Misophonia may also occur in functional neurological disorders (FND) (case 5), potentially with different underpinnings. Although misophonia lacks evidence‐based treatments, behavioral management is preferred over neuropharmacology.^3^ The response of misophonia‐associated tics to therapy beyond trigger avoidance is unclear; however, we guided patients for Comprehensive Behavioral Intervention for Tics (cases 1–4), behavior therapy (case 5), and FND programs (case 6). Recognition will inform clinical and pathophysiological research to develop targeted interventions for this underexplored symptom.

## Author Roles

(1) Research project: A. Conception, B. Organization, C. Execution; (2) Analysis: A. Design, B. Execution, C. Review and critique; (3) Manuscript: A. Writing of the first draft, B. Review and critique.

A.B.: 1C, 2A, 2B, 3A

T.C.G.: 1A, 2B, 2C, 3A, 3B

W.K.L.: 1A, 1B, 2C, 3A, 3B

I.A.M.: 1A, 1B, 2C, 3B

A.E.L.: 1A, 1B, 2C, 3B

C.G.: 1A, 1B, 2C, 3B

## Disclosures

**Ethical Compliance Statement**: We confirm that we have read the journal's position on issues involved in ethical publication and affirm that this work is consistent with those guidelines. The authors confirm that the approval of the University Health Network Research Ethics Board was obtained for this work. Informed consent was obtained for all cases in this case report.

**Funding Sources and Conflicts of Interest**: No specific funding was received for this work. The authors declare that there are no conflicts of interest relevant to this work.

**Financial Disclosures for the Previous 12 Months**: Arjun Balachandar, Talyta Cortez Grippe, Wei Kang Lim, Irene A. Malaty, Anthony E. Lang, and Christos Ganos report no relevant funding sources in the past 12 months.

## Data Availability

The data that support the findings of this study are available from the corresponding author upon reasonable request.
